# First record of the plant bug genus *Paramiridius* Miyamoto & Yasunaga (Heteroptera, Miridae, Mirinae) from Indochina, with descriptions of two new species from Laos

**DOI:** 10.3897/zookeys.546.6335

**Published:** 2015-12-16

**Authors:** Minsuk Oh, Tomohide Yasunaga, Seunghwan Lee

**Affiliations:** 1 Insect Biosystematics Laboratory, Research Institute for Agriculture and Life Sciences, Seoul National University, Seoul 151-921, Republic of Korea; 2 Department of Agricultural Biotechnology, Seoul National University, Seoul 151-921, Republic of Korea; 3 Research Associate, Division of Invertebrate Zoology, American Museum of Natural History, New York; Plant Protection Division, Myanmar Ministry of Agriculture & Irrigation, c/o Japan International Cooperation Agency (JICA), Myanmar Office, #701 Sakura Tower, No. 339, Bogyoke Aung San Road, Kyauktada, Yangon, Myanmar

**Keywords:** *Paramiridius*, new species, taxonomy, key, Indochina, Laos, Heteroptera, Miridae, Mirinae, Mirini

## Abstract

The mirine plant bug genus *Paramiridius*, previously known only from a single Taiwanese species, is reported from Indochinese Laos for the first time and redefined. Two additional species, *Paramiridius indochinensis* and *Paramiridius laomontanus*, are described as new to science. The female genitalic structures of the genus are documented for the first time. Habitus illustrations, figures of male genitalia, and key are provided for all three known *Paramiridius* species.

## Introduction

The mirine plant bug genus *Paramiridius* was proposed by Miyamoto and Yasunaga (1992) to accommodate a single species known only from Taiwan, *Paramiridius tigrinus* Miyamoto & Yasunaga. The genus is readily recognized by the moderate to rather large body with the conventional mirine shape and typical color pattern (yellow or yellowish green dorsum with dark stripes and maculae). However, no subsequent information has been available since the original description.

During recent field investigations undertaken by Seoul National University, 21 specimens, we perceived as belonging to *Paramiridius*, were collected. Upon closer examination, we can confirm that these Lao specimens represent two undescribed species of *Paramiridius*, herein we describe them as new to science. The present discovery also represents a range extension of the genus in Indochina. *Paramiridius* is redefined and diagnosed, and a key to all known species is provided. The female genitalic structures are examined and figured for the first time.

## Materials and methods

All type specimens are deposited in the collection of Insect Biosystematics Laboratory, Research Institute for Agriculture and Life Science, Seoul National University, Korea (SNU). Digital images used in this paper were captured using a Diagnostic Instruments Insight Camera 14.2 Color Mosaic, with a SPOT Insight System. Specimens were dissected and observed under a Leica S8APO stereoscopic microscope.

All measurements (mean and range) are in millimeters. Terminology of the male and female genitalia primarily follows Yasunaga and Schwartz (2007), but some additional terms, such as ‘lateral lobal sclerite’ and ‘median lobal sclerite’ (Fig. 3), are used to indicate the taxonomic characters properly.

## Results

### Genus Paramiridius

Taxon classificationAnimaliaHemipteraMiridae

Miyamoto & Yasunaga

Paramiridius Miyamoto & Yasunaga, 1992: 93 (gen. n.), type species: *Paramiridius tigrinus* Miyamoto & Yasunaga, 1992: 94, original designation; Schuh 1995: 861 (cat.); Kerzhner and Josifov 1999: 136 (cat.).

#### Diagnosis.

**Figure 1.**
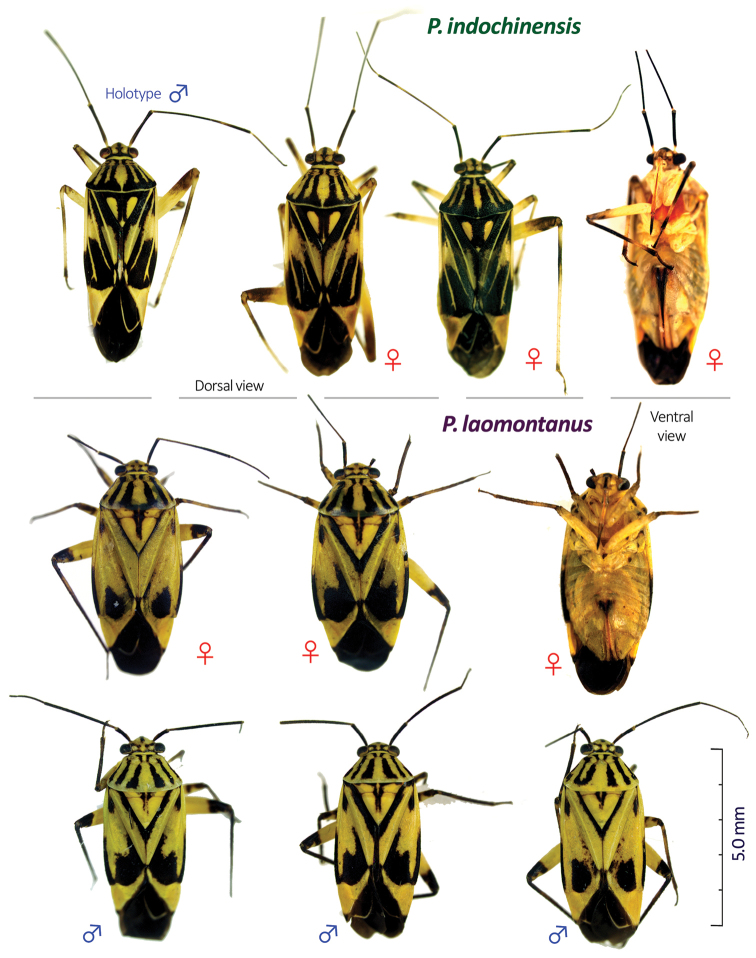
Dorsal and ventral habitus images of two new *Paramiridius* species from Laos.

*Paramiridius* can be distinguished from other known mirine genera by the following combination of characters: moderate to rather large size; sparsely distributed vestiture; weakly shining, matte dorsum with noticeable dark pattern (yellow with dark stripes and maculae as in Fig. 1); generally slender antenna; six or eight dark stripes on pronotum; always wholly darkened mesoscutum; endosoma with a spicule, two (lateral and median) lobal sclerites; apically situated secondary gonopore; posterior wall of bursae with distinct interramal lobe and rather narrowed interramal sclerite; and thick-rimmed sclerotized ring with a developed dorsal labiate plate. For further diagnostic characters, see Miyamoto and Yasunaga (1992).

#### Distribution.

Indochina (Laos), Taiwan.

#### Biology.

Unknown; almost all available specimens were collected using UV light traps. Two females of *Paramiridius laomontanus* were found on *Castanea* sp. (Fagaceae).

#### Discussion.

The original authors (Miyamoto and Yasunaga 1992) mentioned *Paramiridius* is similar in some external characters to two western Palearctic genera, *Miris* Fabricus and *Miridius* Fieber. Nonetheless, the relationships with these genera are now considered only superficial, on the basis of completely different structures exhibited in the male genitalia.

The present work suggests *Paramiridius* is more probably related to *Lygocoris* Reuter, based on sharing the following characters: apically tuberculate phallotheca; presence of a single spicule and apically situated secondary gonopore on endosoma; and similar shape of female sclerotized rings and posterior wall. However, *Paramiridius* is readily distinguished from *Lygocoris* by the unique dark pattern on the dorsum which is nearly matte and glabrous, the different shape of the parameres, and the posterior wall of bursae lacking a lateral lobe (for principal diagnostic characters of *Lygocoris*, see Yasunaga 1991).

There are quite a few mirines superficially similar to *Paramiridius*. To demonstrate more reliable systematic position of the genus, further comprehensive revision is required, including the acquisition of DNA sequence data for representatives of all related genera, a long-run task far beyond the scope of this study.

#### Key to *Paramiridius* species

**Table:** 

1	Basal two-third part of antennal segment II and almost entire scutellum yellowish brown; known only from Taiwan	***tigrinus* Miyamoto & Yasunaga**
–	Antennal segment II wholly infuscate, without pale portions; scutellum mesally with a dark stripe; known from Laos	**2**
2	Body elongate oval, subparallel-sided; dark, mesal stripe on scutellum broad and continuous from base to apex (Fig. 1); all coxae pale, immaculate	***indochinensis* sp. n.**
–	Body nearly ovoid, short; dark, mesal stripe on scutellum narrow, obliterated at apical 1/2–1/3 (Fig. 1); each coxa with a few, dark, small spots	***laomontanus* sp. n.**

### Paramiridius indochinensis sp. n.

Taxon classificationAnimaliaHemipteraMiridae

http://zoobank.org/9EA50EC3-729E-4EB7-B76F-71A77956B484

Figs 1 , 2 , 3 , 4

#### Diagnosis.

**Figure 2.**
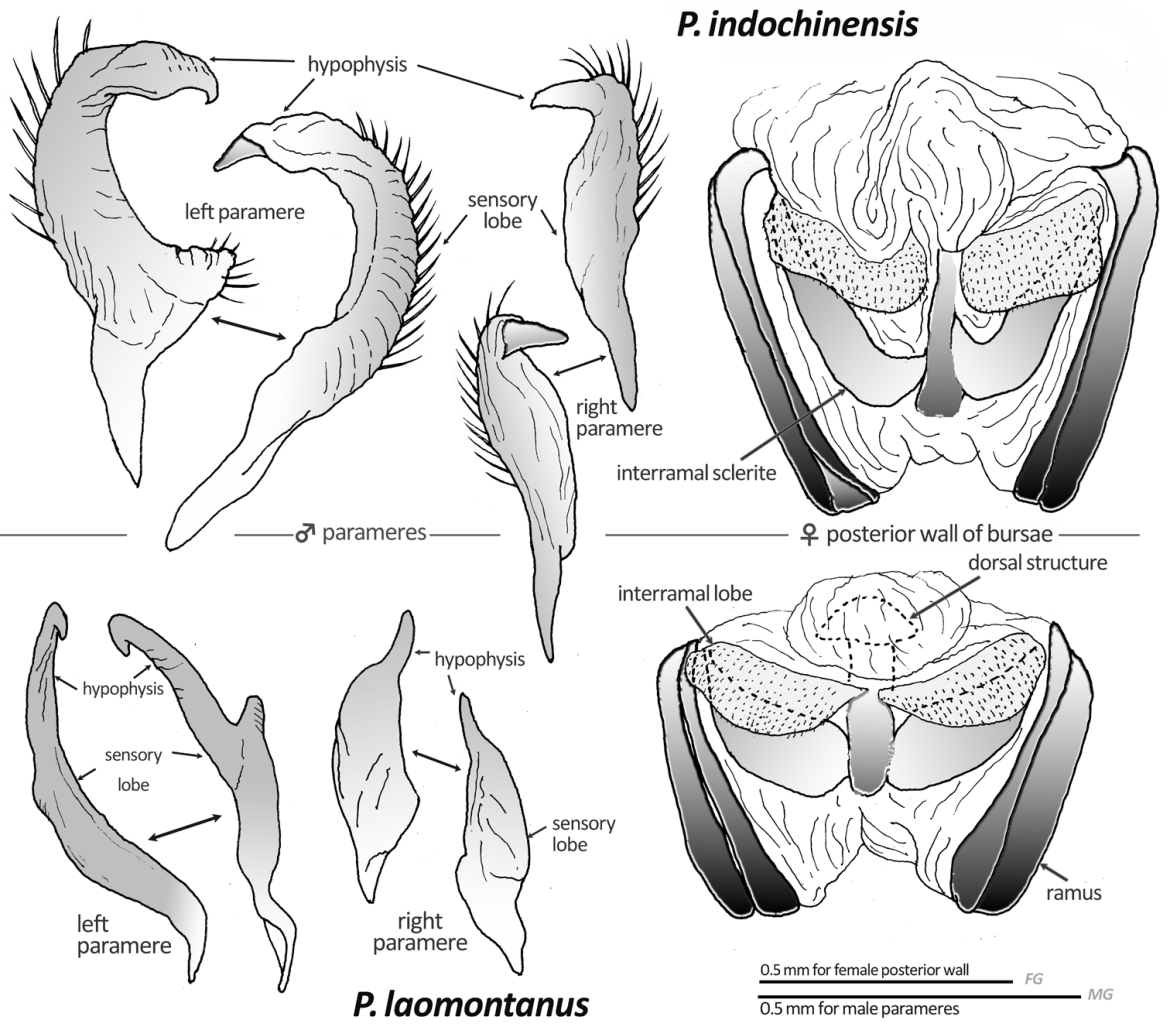
Male parameres and female posterior wall of two new *Paramiridius* species from Laos.

**Figure 3.**
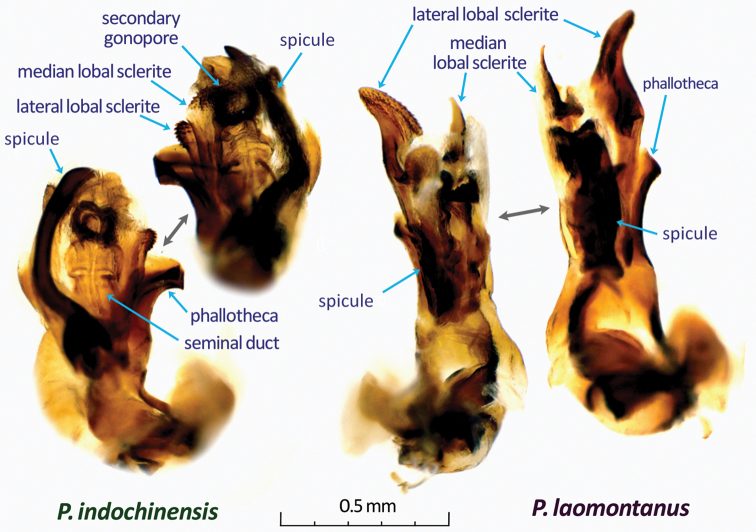
Male endosoma of two new *Paramiridius* species from Laos.

Recognized by the characters given in the key, and the tapered hypophysis of the right paramere (Fig. 2), the developed, curved endosomal spicule (Fig. 3), and the wide, squared interramal lobe (Fig. 2). Most closely related to *Paramiridius tigrinus*, from which this new species can be distinguished by the preceeding diagnostic characters.

#### Description.

*Coloration*: Body generally yellow; dorsum with black-striped patterns (Fig. 1). Head yellow, medially black; eye margin black. Antenna almost entirely dark brown without pale portions. Labium shiny yellowish brown; segment IV darkened. Pronotum yellow, with three pairs of black stripes (each pair fused together posteriorly); and with narrowly yellow posterior margin. Mesoscutum wholly black. Scutellum medially black; lateral part and apex yellow. Hemelytron widely blackish brown, with three pairs of yellow stripes each along claval vein, claval suture and R+M vein (Fig. 1); posterior half of clavus, anterior quarter to half part of corium, anterior two-third of embolium and entire cuneus yellow. Coxa pale yellow; leg yellow; each femur with more or less darkened apical part; each tibia yellow, with a dark, subbasal annulation and darkened apex; all tarsi brown.

*Structure and vestiture*: As in generic description provided by Miyamoto and Yasunaga (1992). Body elongate, parallel-sided; dorsal vestiture generally short, simple, and only sparsely distributed. Head vertical; vertex apparently wider than an eye in dorsal view. Labium reaching middle part of metacoxa.

*Male genitalia* (Figs 2, 3): Similar to type species of the genus, *Paramiridius tigrinus*. Left paramere with a sharp, triangular subbasal protuberance; hypophysis of right paramere developed, claw-like (Fig. 2). Endosomal spicule broad, curved at basal one third, somewhat flattened apically (Fig. 3).

**Figure 4.**
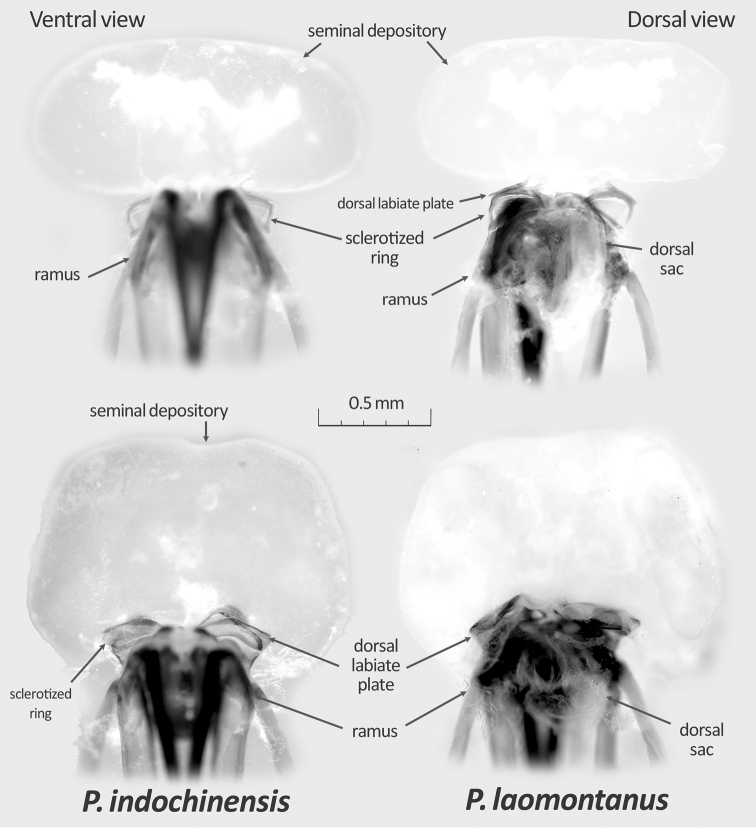
Female bursa copulatrix of two new *Paramiridius* species from Laos.

*Female genitalia* (Figs 2, 4): Bursa copulatrix as in Fig. 4; dorsal labiate plate ventro-medially produced. Interramal lobe wide and squared; interramal sclerite narrowed (Fig. 2).

*Measurements* ♂/♀: Total body length 5.88/ 6.42–7.02; head width across eyes 1.09/ 1.13–1.17; vertex width 0.49/ 0.50–0.52; lengths of antennal segment I–IV 1.25, 3.43, 1.32, ?/ 1.24–1.26, 2.67–2.81, 1.71–1.76, 0.42–0.44; labial length 2.10/ 2.18–2.34; mesal pronotal length including collar 1.23/ 1.29–1.40; basal pronotal width 1.96/ 2.09–2.25; width across hemelytron 2.06/ 2.21–2.43; cuneal length 1.13/ 1.18–1.22; cuneal width 0.72/ 0.81–0.92; lengths of metafemur, tibia and tarsus 3.06, 4.21, 0.75/ 3.08–3.19, 4.38–4.56, 0.73–0.77.

#### Etymology.

Named for its occurrence in the Indochina.

#### Distribution.

Laos (Xiang Khoang Province).

#### Type material.

**Holotype** ♂: **LAOS**: Xiang Khoang Prov., Kham Dist., Phosabous National Protected Area, Namchack Village, [N19°50'57" E103°47'51", 670m alt.], light trap, 2 May, 2015, Oh, 1♂ (SNU). **Paratypes: LAOS**: Same data as for holotype, 3♀♀ (SNU).

### Paramiridius laomontanus sp. n.

Taxon classificationAnimaliaHemipteraMiridae

http://zoobank.org/2B495EF2-9E41-4900-8008-EA6CFB6A1CF4

Figs 1 , 2 , 3 , 4

#### Diagnosis.

Recognized by the characters in the key, and the modified shape of the parameres (Fig. 2), and the well-developed lateral lobe and short spicule on the endosoma (Fig. 3). By these characters, *Paramiridius laomontanus* can be readily distinguished from other congeners.

#### Description.

*Coloration*: Body yellow, often tinged with green, with black maculae and stripes. Head yellow, with paired, symmetrical, dark maculae on vertex; frons with a black stripe medially; clypeus darkened basally. Antenna dark brown to black; extreme bases of segments II and III white. Labium dark brown, except for yellowish segment II. Pronotum greenish yellow, with three pairs of black stripes not reaching pale posterior margin of pronotum. Mesoscutum and scutellum yellow, with symmetrical, dark patterns. Hemelytron pale green or greenish yellow, with variable black patterns as in Fig. 1; inner margin of clavus, and apical half of embolium blackish brown. Coxa pale yellowish brown, with a dark spot basally; leg yellowish brown; pro- and mesofemur with dark brown spots; apex of each femur more or less darkened; all tibiae and tarsi dark brown.

*Structure and vestiture*: Body rather ovoid; dorsal surface with sparsely distributed, simple, pale, short setae. Eye small, contiguous to pronotal collar; vertex wide. Antenna generally slender. Labium comparatively broad, extending to apex of mesocoxa. Pronotum rather tumid, not carinate laterally. Hemelytron shallowly and roughly punctate, almost glabrous.

*Male genitalia* (Figs 2, 3): Parameres glabrous; left paramere slender and nearly straight, with a protuberance at middle and an apically hooked hypophysis; right paramere simple (Fig. 2). Endosoma with a broadened, thin spicule, a tapered median lobal sclerite, and an apically developed, brush-shaped lateral lobal sclerite (Fig. 3).

*Female genitalia* (Figs 2, 4): Bursa copulatrix similar to that of the preceding species, but dorsal labiate plate more developed (Fig. 4). Posterior wall of bursae with rounded interramal sclerite and rather broadened interramal sclerite (Fig. 2).

*Measurements* ♂/♀: Total body length 5.29–5.88/ 5.98–6.53; head width across eyes 1.16–1.26/ 1.22–1.30; vertex width 0.55–0.62/ 0.58–0.66; lengths of antennal segment I–IV 0.78–0.82, 2.10–2.15, 0.97–1.15, 0.66–0.72/ 0.81–0.82, 2.12–2.26, 1.18–1.28, 0.67; labial length 1.49–1.69/ 1.63–1.78; mesal pronotal length including collar 1.17–1.27/ 1.24–1.39; basal pronotal width 1.93–2.14/ 2.13–2.29; width across hemelytron 2.11–2.34/ 2.46–2.74; cuneal length 1.10–1.21/ 1.08–1.20; cuneal width 0.73–0.84/ 0.79–0.93; lengths of metafemur, tibia and tarsus 2.02–2.28, 2.83–3.15, 0.71–0.77/ 2.20–2.57, 3.08–3.37, 0.73–0.77.

#### Etymology.

Named for its occurrence in mountain of Laos.

#### Distribution.

Laos (Xiang Khoang Province).

#### Type material.

**Holotype** ♂: **LAOS**: Xiang Khoang Prov., Kham Dist., Phosabous National Protected Area, Namchack village, [N19°50'57", E103°47'51", 670m alt.], light trap, 2 May, 2015, Oh, 1♂ (SNU). **Paratypes: LAOS**: Xiang Khoang Prov., Kham Dist., Phosabous National Protected Area, Tha Village Middle School, light trap, 1 May, 2015, Oh, 3♂♂ 3♀♀ (SNU). Same data as for holotype, 8♂♂ (SNU). Namchack village, [N19°50'57", E103°47'51", 670m alt.], sweeping *Castanea* sp., 2 May, 2015, Oh, 2♀♀ (SNU).

### Paramiridius tigrinus

Taxon classificationAnimaliaHemipteraMiridae

Miyamoto & Yasunaga

Paramiridius tigrinus Miyamoto & Yasunaga, 1992: 94 (sp. n.); Schuh 1995: 861 (cat.); Kerzhner and Josifov 1999: 136 (cat.).

#### Diagnosis.

Recognized by the characters mentioned in the key, and the broader yellow posterior margin of the pronotum, the slender, blunt-tipped hypophysis of the right paramere, and the straight endosomal spicule (See Miyamoto and Yasunaga 1992).

#### Distribution.

Taiwan.

## Supplementary Material

XML Treatment for Paramiridius

XML Treatment for Paramiridius indochinensis

XML Treatment for Paramiridius laomontanus

XML Treatment for Paramiridius tigrinus
